# Pediatric Tuberculosis Care Outcomes in a Tertiary Care Center, France, 2014–2024

**DOI:** 10.3201/eid3209.260600

**Published:** 2026-09

**Authors:** Diane Franco, Luc Panetta, Florence Ader, Antoine Ouziel, Yves Gillet, Côme Horvat

**Affiliations:** Hospices Civils de Lyon, Lyon, France (D. Franco, L. Panetta, F. Ader, A. Ouziel, Y. Gillet, C. Horvat); Centre Hospitalier Universitaire Sainte-Justine, Montreal, Quebec, Canada (L. Panetta).

**Keywords:** tuberculosis, Pediatric tuberculosis, treatment outcomes, WHO definitions, quality of care, treatment adherence, dosing adequacy, low-incidence setting, high-income country, bacteria, bacterial infections, tuberculosis and other mycobacteria, France

## Abstract

We evaluated pediatric tuberculosis treatment outcomes in a tertiary care center in France during 2014–2024 by using updated World Health Organization definitions. Among 123 children, 60.9% were successfully treated. Unfavorable outcomes reflected suboptimal adherence, dosing errors, and incomplete follow-up, highlighting the need for implementation and evaluation of optimized care processes.

Pediatric tuberculosis (TB) remains a major global health concern; an estimated 1.2 million new cases and 174,300 deaths were reported in 2024 (*1*). Treatment regimens are well-standardized and updated by the World Health Organization (WHO) (*2*). However, research and implementation efforts predominantly focus on high-incidence countries. Data from pediatric cohorts in high-income, low-incidence settings remain scarce, reporting overall success rates around 90% (*3*,*4*), but provide very limited insights into the quality of care, treatment pathways, and potential barriers. We describe TB treatment outcomes in a tertiary pediatric center in France over a 10-year period by using the updated WHO treatment–outcome definitions as a standardized framework.

We conducted a retrospective cohort study at a tertiary pediatric hospital in Lyon, France, including all patients <18 years of age treated for TB during January 1, 2014–December 31, 2024. TB diagnosis was consistent with WHO guidelines (*2*). To achieve comprehensive case identification, we cross-matched 4 institutional databases, including microbiology, pharmacy, administrative records, and pediatric infectious disease consultations, and then conducted a systematic manual review of each medical record.

We collected data on demographics, signs and symptoms, treatment, and outcomes by using standardized definitions (*2*,*5*–*7*). We classified treatment outcomes according to WHO definitions as favorable (cured or treatment completed) or unfavorable (treatment failure, lost to follow-up, or death) (Appendix Table) (*8*). To further elucidate the care process, we captured secondary analytic outcomes. Those outcomes included dosing adequacy, assessed according to WHO-recommended weight-based drug dosages and, when available, therapeutic drug monitoring for rifampin or isoniazid; treatment nonadherence (intake <80% or treatment interruption for >2 consecutive weeks (*6*); follow-up nonadherence (any missed scheduled visit); microbiological failure; treatment prolongation; modification of the initial regimen (excluding adaptations on the basis of drug susceptibility); and treatment-related adverse events (*7*). 

Among 123 children with TB, 13 were not evaluated according to WHO outcome definitions because they were transferred to another facility. Of the 110 evaluable children, 67 (60.9%) had favorable treatment outcomes and 43 (39.1%) had unfavorable treatment outcomes, including 34 with treatment failures, 9 who were lost to follow-up, and 2 with documented microbiological failure (Figure). Treatment failure was related to radiological failure (32.6%, n = 14), clinical failure (16.3%, n = 7), or treatment nonadherence or underdosing (23.3%, n = 10). Underdosing was documented in 28 (25.4%) children, 11 (10%) with subtherapeutic drug concentrations and 17 (15.5%) with subrecommended weight-based dosing. Overdosing was documented in only 1 child. Therapeutic nonadherence was reported in 17 (15.5%) children, and follow-up nonadherence was reported in 67 (60.9%) children. Thirteen (11.8%) children experienced adverse events, including hepatotoxicity (8.2%, n = 9), agranulocytosis (2.7%, n = 3), peripheral neuropathy (0.9%, n = 1), and optic toxicity under ethambutol (0.9%, n =1). Nearly half of the adverse events (46%, n = 6) led to permanent discontinuation of the implicated drug.

**Figure:**
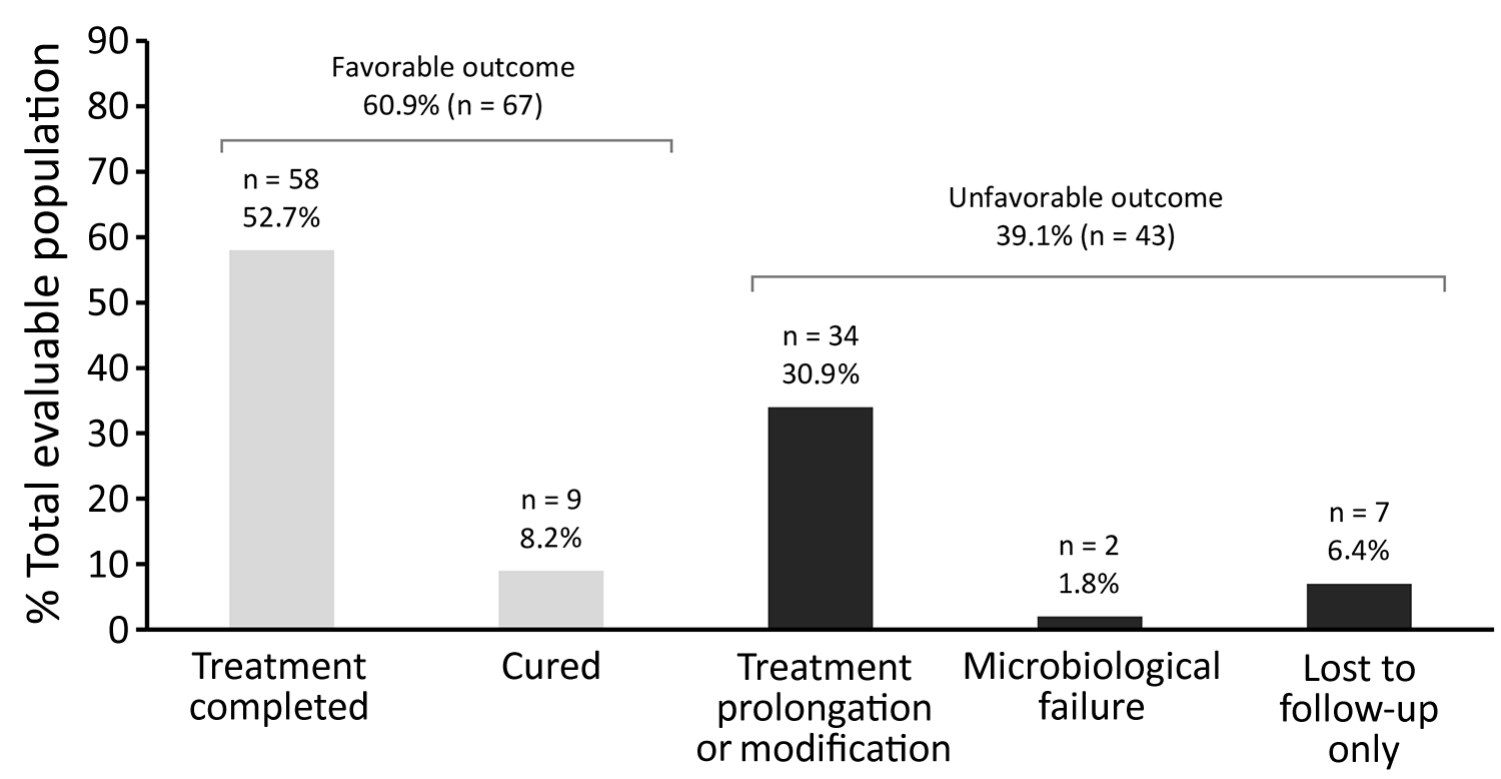
Distribution of pediatric tuberculosis care outcomes in a tertiary care center, France, 2014–2024. Light gray bars indicate favorable outcomes and black bars unfavorable outcomes, as defined by the World Health Organization. Percentage values reflect the percentage of the total evaluable population (n = 110).

Of the 110 evaluable children, the median age was 5 years; 61 (55.5%) were male and 49 (45.5%) female (Table). Malnutrition was documented in 10 (9.1%) children, and 4 (3.6%) children were co-infected with HIV. Isolated thoracic disease was documented in 78 (70.9%) children, including 50 children with severe forms of the disease. Extrapulmonary TB was reported in 32 (29.1%) children. We obtained microbiological confirmation from 58 (52.7%) children and documented index cases in 43 (39.1%) children. Five (4.5%) TB strains were resistant: 3 resistant to isoniazid, 1 resistant to ethambutol, and 1 multidrug-resistant. Most (57.3%, n = 63) children were treated with the standard 6–month regimen; only 1 received the 4–month regimen, and 13 (11.8%) children with severe disease required prolonged treatment for 9–12 months (*2*).

**Table:** Population characteristics of pediatric patients with TB, France, 2014–2024*

Characteristics	No. (%) patients		
	Total, n = 110	Favorable outcome, n = 67	Unfavorable outcome, n = 43
Age group, y			
<1	11 (10.0)	7 (10.4)	4 (9.3)
1–5	46 (41.8)	28 (41.8)	18 (41.9)
5–10	20 (18.2)	13 (19.4)	7 (16.3)
10–18	33 (30)	19 (28.4)	14 (32.5)
Sex			
M	61 (55.5)	40 (59.7)	21 (48.8)
F	49 (45.5)	27 (41.3)	22 (51.2)
Underlying conditions			
Malnutrition	10 (9.1)	6 (9.0)	4 (9.3)
HIV co-infection	4 (3.6)	2 (3.0)	2 (4.7)
Extrapulmonary tuberculosis complications			
Isolated thoracic TB	78 (70.9)	52 (77.6)	26 (60.5)
Peripheral lymphadenitis	18 (16.4)	8 (7.3)	10 (9.1)
Central nervous system TB	6 (5.5)	4 (3.6)	2 (1.8)
Osteoarticular TB	6 (5.5)	4 (3.6)	2 (1.8)
IRIS	8 (7.3)	2 (3.0)	6 (14.0)
BCG vaccination	45 (40.9)	36 (53.7)	9 (20.9)
Microbiologic characteristics			
Microbiological confirmation	58 (52.7)	31 (46.3)	27 (62.8)
Identified index case	43 (39.1)	32 (47.8)	11 (25.6)
Drug-resistant strain	5 (4.5)	2 (3.0)	3 (7.0)
Initial treatment characteristics			
Planned treatment duration, mo			
<6	64 (58.2)	55 (82.1)	9 (20.9)
>6	13 (11.8)	12 (17.9)	1 (2.3)
Initial treatment regimen			
Standard 4-drug regimen: HRZE	62 (56.4)	38 (56.7)	24 (55.8)
Standard 3-drug regimen: HRZ	46 (41.8)	27 (40.3)	19 (44.2)
Other treatment regimen	2 (1.8)	2 (3.0)	0

* BCG, Bacille Calmette-Guérin; HRZ, isoniazid, rifampin, and pyrazinamide; HRZE, isoniazid, rifampin, pyrazinamide, and ethambutol; IRIS, immune reconstitution inflammatory syndrome; TB, tuberculosis

By using standardized WHO definitions in a high-income, low-incidence setting, we found nearly 40% of children had unfavorable treatment outcomes. Care-process gaps were frequent: 1 in 4 children were underdosed, treatment nonadherence was documented in 1 in 6 children, and ≈66% of children had suboptimal follow-up attendance. Those findings underscore that many unfavorable outcomes reflect elements of care that can be improved rather than intrinsic disease severity or care access.

Our results are in striking contrast with reported success rates ≈90% in similar settings, largely on the basis of heterogeneous or earlier outcome definitions (*3*,*4*), highlighting how updated WHO definitions go beyond death or microbiological failure to capture suboptimal treatment processes. Furthermore, the low relevance of the cured category, because of limited microbiological documentation in children (*9*), reinforces the value of treatment completed as a more meaningful indicator in pediatric TB.

Although the retrospective and single-center design of our study limits its generalizability, its decade-long timespan, exhaustive case identification, and rigorous application of standardized WHO definitions strengthen our findings. Similar concerns have been raised in other high-income settings, where organizational models of TB care have influenced treatment outcomes (*10*).

In conclusion, applying updated WHO definitions revealed a high proportion of unfavorable outcomes in a high-income, low-incidence setting. This revelation supports the implementation and evaluation of optimized care processes focused on treatment adherence, accurate weight-based drug dosing, and structured follow-up.

Appendix Additional information about pediatric tuberculosis care outcomes in a tertiary care center, France, 2014–2024.
