# Supplementary material for: Pediatric Tuberculosis Care Outcomes in a Tertiary Care Center, France, 2014–2024
Source: Emerg Infect Dis. 2026 Sep;32(9):1511–3. doi: 10.3201/eid3209.260600 (PMC13555715; doi:10.3201/eid3209.260600)
Supplement: Supplementary file 1 [file 26-0600-Techapp-s1.pdf]

Article DOI: https://doi.org/10.3201/eid3209.260600

EID cannot ensure accessibility for supplementary materials supplied by authors. Readers who have difficulty accessing supplementary content should contact the authors for assistance.

Pediatric Tuberculosis Care Outcomes in a Tertiary Care Center, France, 2014–2024

Appendix

Appendix Table. Updated WHO definitions of tuberculosis treatment outcomes*

Outcome	Definition
Treatment failed	A patient whose treatment regimen needed to be terminated or permanently changed† to a new regimen or treatment strategy.
Cured	A patient with pulmonary TB with bacteriologically confirmed TB at beginning of treatment who completed treatment recommended by the national policy, with evidence of bacteriological response‡ and no evidence of failure.
Treatment completed	A patient who completed treatment recommended by the national policy but whose outcome does not meet the definition for cure or treatment failure.
Died	A patient who died§ before starting treatment or during the course of treatment.
Lost to follow-up	A patient who did not start treatment or whose treatment was interrupted for 2 consecutive months or more.
Not evaluated	A patient for whom no treatment outcome was assigned¶
Treatment success	The sum of all patients cured and treatment completed.

*DR-TB, drug-resistant tuberculosis; DS-TB, drug-susceptible tuberculosis; TB, tuberculosis.
†Reasons for the change include: no clinical response or no bacteriological response, or both (see note '‡'); adverse drug reactions; or evidence of additional drug-resistance to medicines in the regimen.
‡"Bacteriological response" refers to bacteriological conversion with no reversion. "Bacteriological conversion" describes a situation in a patient with bacteriologically confirmed tuberculosis where at least 2 consecutive cultures (for drug-resistant tuberculosis and drug-susceptible tuberculosis) or smears (for drug-susceptible tuberculosis only) taken on different occasions at least 7 d apart are negative. "Bacteriological reversion" describes a situation where at least two consecutive cultures (for drug-resistant tuberculosis and drug-susceptible tuberculosis) or smears (for drug-susceptible tuberculosis only) taken on different occasions at least 7 d apart are positive either after the bacteriological conversion or in patients without bacteriological confirmation of tuberculosis.
§Patient died for any reason.
¶This includes cases "transferred out" to another treatment unit and whose treatment outcome is unknown; however, it excludes those lost to follow-up.
